# Supplementary material for: A Systematic Review Exploring Dietary Behaviors, Psychological Determinants and Lifestyle Factors Associated with Weight Regain After Bariatric Surgery
Source: Healthcare (Basel). 2024 Nov 11;12(22):2243. doi: 10.3390/healthcare12222243 (PMC11594053; doi:10.3390/healthcare12222243)

Supplementary Materials

1.1 PRISMA 2020 flow diagram for new systematic reviews which included searches of databases and registers only

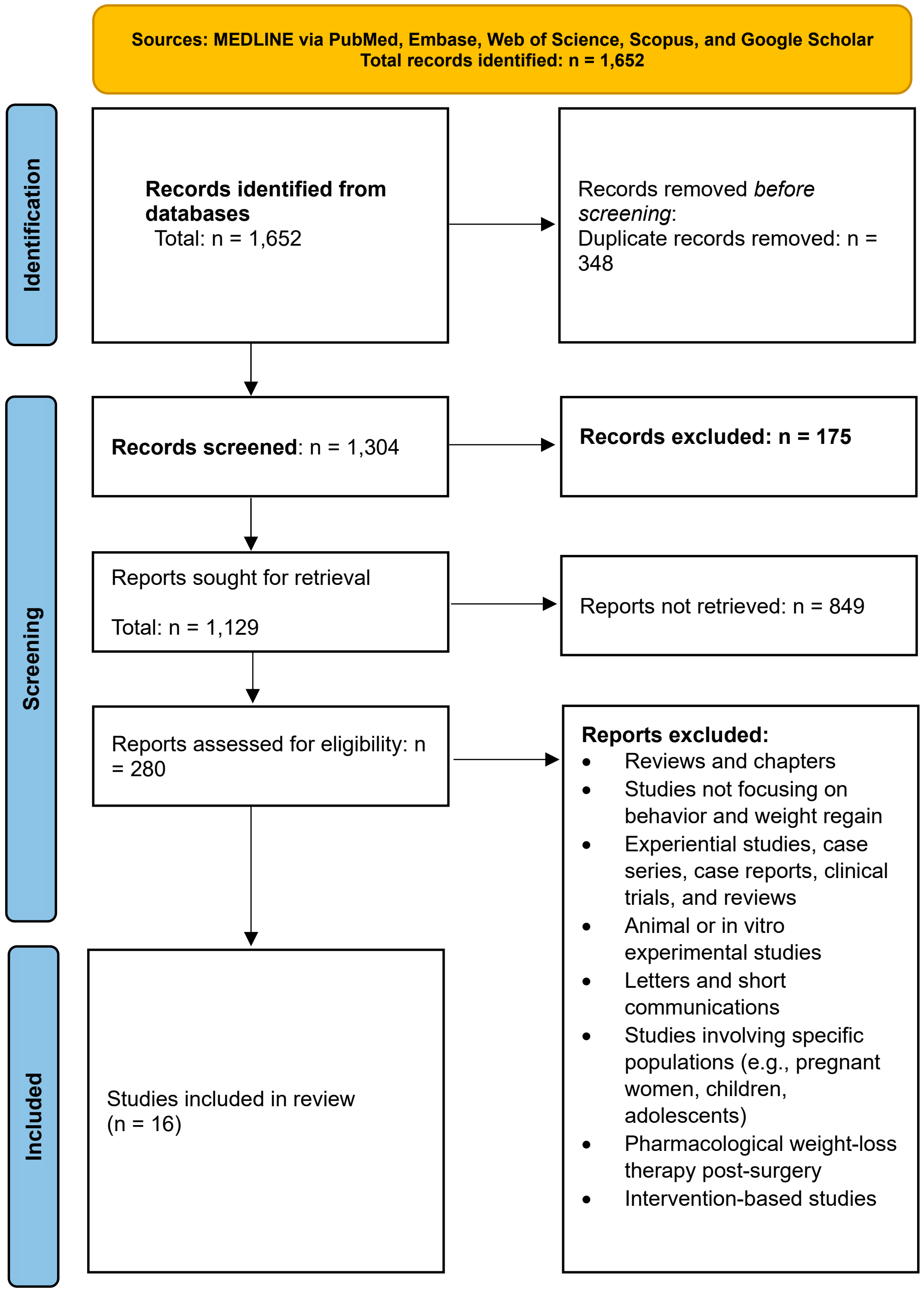

1.2 Table of the search strategy:

Database	Search Terms	Filters Applied	Data of Search	Records Identified
PubMed	("bariatric surgery" OR "weight loss surgery") AND ("weight regain" OR "weight gain") AND ("dietary behavior" OR "psychological")	Humans, English language, Last 10 years	July 10, 2023,	450
Embase	("bariatric surgery" OR "obesity surgery") AND ("weight regain" OR "postoperative weight gain") AND ("lifestyle" OR "diet")	Humans, English language, Adults	July 10, 2023,	380
Web of Science	("weight regain" OR "weight gain") AND ("bariatric surgery") AND ("psychological factors" OR "dietary patterns")	English language, Peer-reviewed	July 11, 2023,	290
Scopus	TITLE-ABS-KEY("weight regain" AND "bariatric surgery") AND ("psychology" OR "eating habits")	English language, Reviews excluded	July 11, 2023,	320
Google Scholar	"Weight regain after bariatric surgery" AND "behavioral factors"	last 10 years	July 12, 2023,	212

1.3 Table of Risk Bias Score Assessments:

Reference and Year	Design/Time Since Surgery	Risk of Bias Score	Behavioral Factors Category.
Althumiri, 2024 [15]	Cross-Sectional <6 months	7	Dietary non-adherence and eating patterns & Psychological Determinants & Lifestyle behaviors
Althumiri, 2023 [14]	Cross-Sectional <6M.	8	Dietary non-adherence and eating patterns & Lifestyle behaviors
Amundsen, 2018 [16]	Cross-Sectional - >1Y.	8	Dietary non-adherence and eating patterns & Lifestyle behaviors
Berino, 2022 [17]	Cross-Sectional >2Y and <10Y	6	Dietary non-adherence and eating patterns & Lifestyle behaviors
Conceição, 2014 [18]	Cross-Sectional (6m, 1y, 2y)	7	Dietary non-adherence and eating patterns & Psychological Determinants
Da Silva, 2016 [12]	Cohort Study >2Y.	7	Dietary non-adherence and eating patterns
Dos Rodrigues, 2021 [9]	Cross-Sectional >2Y.		Dietary non-adherence and eating patterns & Lifestyle behaviors
Freire, 2021 [19]	Cross-Sectional 1. 2Y; 2.7-14Y	7	Dietary non-adherence and eating patterns & Psychological Determinants
King, 2020 [13]	Cohort Study	8	Dietary non-adherence and eating patterns & Psychological Determinants & Lifestyle behaviors
McInnis, 2022 [20]	Cross-Sectional >2Y.	7	Dietary non-adherence and eating patterns
Miller-Matero, 2024 [21]	Cross-Sectional >2Y.	8	Dietary non-adherence and eating patterns & Lifestyle behaviors
Monpellier, 2019 [22]	Cohort Study at 15, 24, 36 and 48 M.	8	Dietary non-adherence and eating patterns & Psychological Determinants
Nicanor-Carreón,2023 [23]	Cross-Sectional 2–10Y.	7	Dietary non-adherence and eating patterns
Romagna, 2021 [24]	Cross-Sectional 1. <5Y ; 2.>5Y	7	Lifestyle behaviors
Tolvanen, 2023 [25]	In-depth semi-structured individual interviews	6	Dietary non-adherence and eating patterns & Psychological Determinants & Lifestyle behaviors
Vieira, 2019 [26]	Cross-Sectional >2Y.	7	Dietary non-adherence and eating patterns

1.4 JBI CRITICAL APPRAISAL CHECKLIST FOR ANALYTICAL CROSS SECTIONAL STUDIES

Reviewer__________ Date_______

Author_______ Year ___ Record Number______

	Yes	No	Unclear	Not applicable
1. Were the criteria for inclusion in the sample clearly defined?	☐	☐	☐	☐
2. Were the study subjects and the setting described in detail?	☐	☐	☐	☐
3. Was the exposure measured in a valid and reliable way?	☐	☐	☐	☐
4. Were objective, standard criteria used for measurement of the condition?	☐	☐	☐	☐
5. Were confounding factors identified?	☐	☐	☐	☐
6. Were strategies to deal with confounding factors stated?	☐	☐	☐	☐
7. Were the outcomes measured in a valid and reliable way?	☐	☐	☐	☐
8. Was appropriate statistical analysis used?	☐	☐	☐	☐

Overall appraisal: Include ☐ Exclude ☐ Seek further info ☐

Comments (Including reason for exclusion)

___

___

___

1.5 **Interacting Factors Contributing to Weight Regain After Bariatric Surgery**

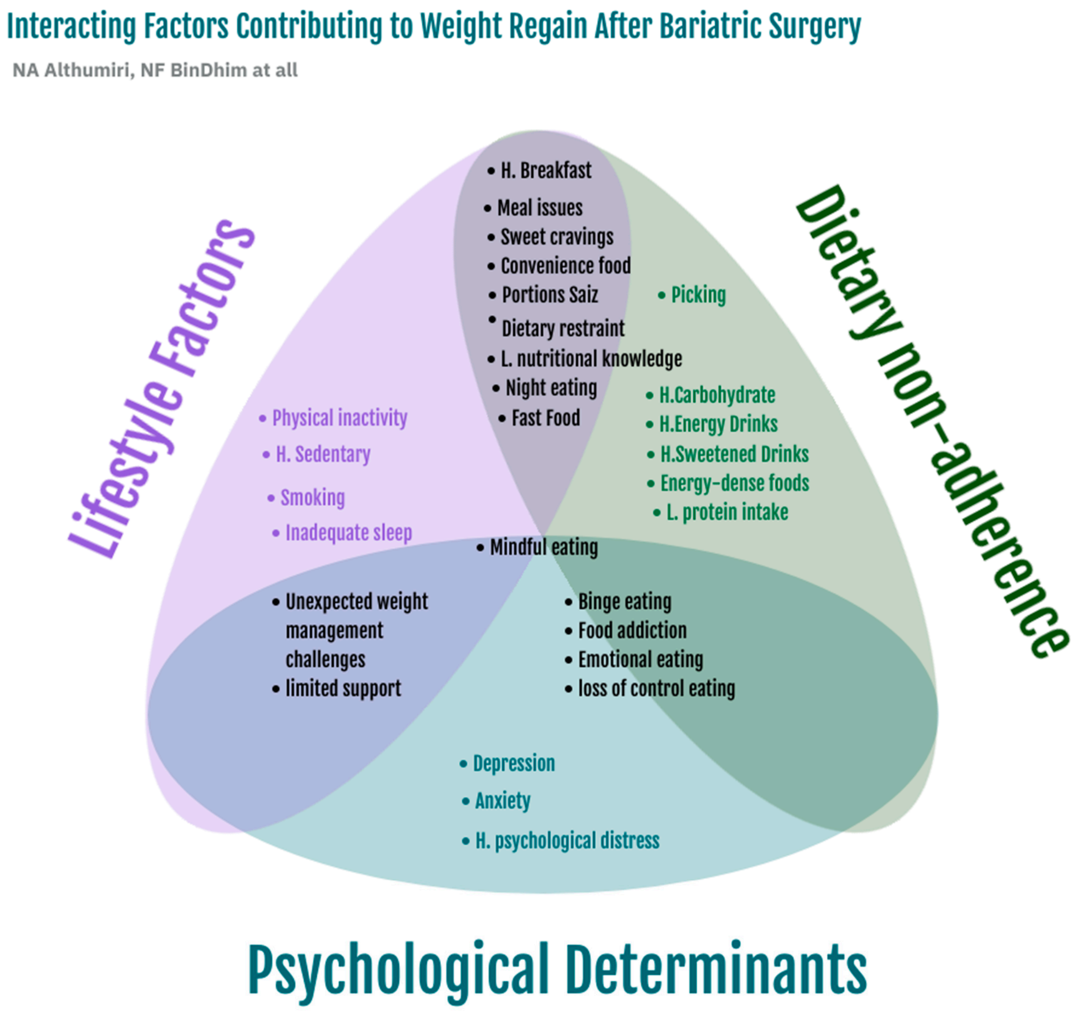

Supplement: Supplementary file 1 [file healthcare-12-02243-s001.zip › healthcare-3287967-supplementary.pdf]
